# Improved serological detection of rheumatoid arthritis: a highly antigenic mimotope of carbonic anhydrase III selected in a murine model by phage display

**DOI:** 10.1186/s13075-015-0685-3

**Published:** 2015-06-23

**Authors:** Galber Rodrigues Araujo, Emília Rezende Vaz, Patricia Tiemi Fujimura, João Eurico Fonseca, Lucélia Maria de Lima, Helena Canhão, Gabriela Venturini, Karina Helena Morais Cardozo, Valdemir Melechco Carvalho, Marcelo Henrique Napimoga, Luiz Ricardo Goulart, João Gonçalves, Carlos Ueira-Vieira

**Affiliations:** Laboratory of Nanobiotechnology, Institute of Genetics and Biochemistry, Federal University of Uberlândia, Uberlândia, MG Brazil; iMed.UL – Research Institute for Medicines and Pharmaceutical Sciences, Faculty of Pharmacy, University of Lisbon, Lisbon, Portugal; Rheumatology Research Unit, Institute of Molecular Medicine, Lisbon, Portugal; Rheumatology Department, Santa Maria Hospital, Lisbon Academic Medical Center, Lisbon, Portugal; Laboratory of Genetics and Molecular Cardiology, Heart Institute, University of São Paulo Medical School, São Paulo, SP Brazil; Fleury group, São Paulo, SP Brazil; Laboratory of Immunology and Molecular Biology, São Leopoldo Mandic Institute and Research Center, Campinas, SP Brazil; Department of Medical Microbiology and Immunology, University of California Davis, Davis, CA USA; IMM – Institute of Molecular Medicine, Lisbon, Portugal

## Abstract

**Introduction:**

Rheumatoid arthritis (RA) is a chronic inflammatory autoimmune disease that affects around 1 % of the human population worldwide. RA diagnosis can be difficult as there is no definitive test for its detection. Therefore, the aim of this study was to identify biomarkers that could be used for RA diagnosis.

**Methods:**

Sera from a collagen-induced arthritis mouse model were used to select potential biomarkers for RA diagnosis by phage display technology. In silico and in vitro analyses were performed to characterize and validate the selected peptides. Samples were classified into three groups: RA; two other immune-mediated rheumatic diseases (systemic lupus erythematosus (SLE) and ankylosing spondylitis (AS)); and healthy controls (HC). Enzyme-linked immunosorbent assay (ELISA) was carried out to determine antibody levels, and diagnostic parameters were determined by constructing receiver operating characteristic curves. Mass spectrometry and Western blot were performed to identify the putative autoantigen that was mimicked by a highly reactive mimotope.

**Results:**

After three rounds of selection, 14 clones were obtained and tested for immunoreactivity analysis against sera from RA and HC groups. The phage-fused peptide with the highest immunoreactivity (M12) was synthesized, and was able to efficiently discriminate RA patients from SLE, AS and HCs (*p* < 0.0001) by ELISA. The specificity and sensitivity of anti-M12 antibodies for RA diagnosis were 91 % and 84.3 %, respectively. The M12 peptide was identified as one that mimics a predicted antigenic site of the carbonic anhydrase III (CAIII) protein, a ubiquitous biomarker that has been identified in patients with other diseases.

**Conclusion:**

M12 is the first peptide associated with the CAIII protein that may be used as an antigen for antibody detection to aid in RA diagnosis with high sensitivity and specificity.

## Introduction

Rheumatoid arthritis (RA), the most common inflammatory autoimmune disease, affects 0.8 % of the adult population worldwide [1]. RA diagnosis is largely a clinical one, relying, particularly in the early stages, on the history and examination of the patient, with tests (blood or imaging) sometimes helping to confirm the diagnosis [2]. Serological support to diagnosis has, up to now, been restricted to the determination of rheumatoid factor (RF) and anticitrullinated peptide antibodies (ACPAs), where assays using cyclic citrullinated peptides (CCP) as antigen for ACPA detection have gained wide acceptance [3]. RF presents higher sensitivity as compared with antiCCP antibodies for established disease, with a relatively low specificity. In fact, the RF antibody is not specific for RA due to crossreactivity with many other inflammatory diseases, as well as in elderly healthy individuals [4]. ACPAs are considered a valuable serological biomarker for RA [5] and the diagnostic performance of different generations of CCPs (CCP1, CCP2 and CCP3) have been evaluated in many different studies [6–8]. Differences in cut-off values, specificities and sensitivities exist between the three different generations and also between different assays used for antibody detection. However, antiCCP2 showed better performance characteristics with values of sensitivity ranging from 41 % to 92.2 % and specificity ranging from 65 % to 100 % [9]. At present, the detection of antibodies against CCP2 by enzyme-linked immunosorbent assay (ELISA) is the most widely used assay in studies involving ACPAs worldwide. The combination of RF and antiCCP2 assays demonstrate a positive predictive value close to 100 %, which is much higher than the value of either of the tests alone [10]. The presence of RF and antiCCP has been associated with progressive and destructive disease [11, 12]. Seronegativity in both early and established RA remains a major limitation of these two biomarkers, highlighting the need for new complementary markers that could improve diagnostic sensitivity [13]. Because of the low sensitivity or specificity of the current serological tests, the quest for new efficient auxiliary biomarkers in RA is of clinical relevance.

Animal models of arthritis have contributed to the overall knowledge on RA physiopathology and to the identification of important mediators of inflammation. The collagen-induced arthritis (CIA) mouse model has proven to be a valuable experimental model for inflammatory RA studies [14–17]. After immunization with type II collagen (CII), DBA/1 J mice develop a severe polyarthritis mediated by an autoimmune response that shares many features with human RA [18].

With the goal of identifying new clinically useful biomarkers for RA, we have explored the CIA mouse model and phage display (PD) technology to isolate peptides that can mimic RA autoantigens. PD technology has been widely used by our group and others to screen targeting peptides in drug discovery and biomarker selection, and has been highly effective in discovering peptides with affinities to virtually any target [19–24]. Short peptide sequences selected by PD libraries with high affinity to antibodies, receptors or proteins may present potential applications in diagnostics or therapeutic kits and vaccines [25, 26]. Using the cDNA PD library for autoantigen selection, were recently identified novel autoantibodies in early and seronegative RA patients with sensitivities ranging from 2 % to 29 %, and specificities ranging from 95 % to 100 %. These autoantibodies can be found in 44 % to 67 % of ACPA-negative RA patients [27].

This investigation describes the identification of a short peptide selected by PD technology against sera from CIA mice. This short peptide was characterized by in silico and in vitro strategies, and further tested as a potential biomarker for RA diagnosis in comparison to other rheumatic diseases and healthy controls. Its predicted antigenic epitope target was deduced by mass spectrometry (MS) and Western blot analyses.

## Methods

### Study subjects

For this study, we used serum samples from patients who fulfilled the RA diagnostic criteria according to the 2010 classification [28]. The demographic and laboratory characteristics of all studied subjects are presented in Table 1. Well-characterized serum samples used in this study were requested from Biobanco-IMM, Lisbon Academic Medical Center, Lisbon, Portugal. Samples from healthy subjects who did not have any arthritis symptoms were used as the healthy control (HC) group. To provide data on assay specificity, samples from patients with other rheumatic diseases (nonRA) were also analyzed. The subjects enrolled in this study were classified into three groups: RA, 172 patients (151 females and 21 males, mean age 53.7 ± 11.2 years); HC, 113 subjects (79 females and 34 males, mean age 58.8 ± 8.7 years); and nonRA, 32 patients with other immune-mediated rheumatic diseases that consisted of 19 systemic lupus erythematosus (SLE) and 13 ankylosing spondylitis (AS) patients. For RA patients, data recorded were age at disease diagnosis, disease duration, medication status, presence of RF and antiCCP, erythrocyte sedimentation rate (ESR), levels of C-reactive protein (CRP), and tender and swollen joint counts obtained at the time of blood sample collection. Blood samples were allowed to clot and then centrifuged at 4,000 rpm for 10 minutes. All sera samples were filtrated with a microcell filter (0.22 μm) to eliminate red blood cell fragments and bacteria, and then frozen at −80 °C immediately until analysis. The parts of the study which involved human subjects was approved by the ethics committees of the Centro Hospitalar Lisboa Norte, Hospital de Santa Maria and the Hospital Garcia de Orta, Lisbon, Portugal, and conducted in accordance with the regulations governing clinical trials such as the Declaration of Helsinki (2008). An informed consent form was signed by all subjects enrolled in this study before any protocol procedure was carried out.

**Table 1:** Demographic and laboratory characteristics of the studied population

	RA	HC	SLE	AS
Age (years; mean ± SD)	53.7 ± 11.2	58.8 ± 8.7	55.7 ± 5.7	49.5 ± 7.5
Gender (female/male)	151/21	79/34	16/3	4/9
Caucasian (n (%))	146 (85 %)	104 (92 %)	18 (95 %)	11 (85 %)
Disease duration (years; mean ± SD)	8.1 ± 8.4		9.2 ± 6.5	12 ± 9.1
Rheumatoid factor positive (n)	102 (59.3 %)		NA	NA
CRP (mg/dl; mean ± SD)	2.4 ± 1.1		3.1 ± 0.9	2.7 ± 0.8
ESR (mm/hour; mean ± SD)	32 ± 26		33.4 ± 19.5	NA
AntiCCP positive (n)	75 (43.6 %)		NA	NA
Biologic therapy (n in treatment)	62 (36 %)		4 (21 %)	3 (23 %)
NSAIDs (n in treatment)	127 (74 %)		9 (47 %)	5 (38 %)

CRP value ≥0.8 mg/dl is considered elevated. ESR value ≥15 mm/hour is considered elevated. *antiCCP* anticyclic citrullinated peptide antibodies, *AS* ankylosing spondylitis, *CRP* C-reactive protein, *ESR* erythrocyte sedimentation rate, *HC* healthy control, *NA* not available, *NSAIDs* nonsteroidal anti-inflammatory drugs, *RA* rheumatoid arthritis, *SD* standard deviation, *SLE* systemic lupus erythematosus

### CIA mice

A total of twelve male DBA/1 J mice (8–10 weeks old) were housed at the animal facility of the Federal University of Uberlândia (Uberlândia-MG, Brazil) where they fed ad libitum. This study was carried out in strict accordance with the recommendations contained in terms for the use of animals in research and teaching of the Federal University of Uberlândia (Uberlândia, MG, Brazil), in compliance with the National Guidelines, as set forth by the Institutional Animal Care (Law number 11.794, 2008). The study protocol was approved by the Animal Ethics Committee from the Federal University of Uberlândia (UFU) under the number CEUA/UFU 059/10.

### Arthritis induction and assessment of CIA mice

Arthritis was induced in the isogenic murine model DBA/1 J (males; 8–10 weeks old) as described previously with some modifications [18]. Briefly, a commercial bovine CII (Becton Sigma-Aldrich, St. Louis, MO, USA) emulsified in complete Freund’s adjuvant (CFA) (Sigma-Aldrich) was used for mice immunization. DBA/1 J mice (n = 9) were immunized by intradermal injections at the base of the tail with 100 μg bovine CII. For the control group, mice (n = 3) were immunized with phosphate-buffered saline (PBS) in CFA. Mice were boosted at day 30 with 50 μg bovine CII in CFA. After immunization, mice were examined twice a week when paw edema was measured using manual calipers and arthritis signs were visually scored by evaluating joint inflammation, using an established scoring system from 0 to 4 [29], where 0 = no evidence of erythema and swelling, 1 = erythema and mild swelling confined to the tarsals or ankle joints, 2 = erythema and mild swelling extending from the ankle to the tarsals, 3 = erythema and moderate swelling extending from the ankle to metatarsal joints, 4 = erythema and severe swelling encompass the ankle, foot and digits, or ankylosis of the limb. Around 60 days after immunization, mice presenting acute arthritis (score 4) were sacrificed under anesthesia. Blood (for sera extraction) and inflamed joints were then extracted for peptide selection through PD technology.

### Total protein extraction from inflamed joints of CIA mice

Inflamed joints were removed from CIA mice, macerated with liquid nitrogen and suspended in extraction buffer (20 mM Tris–HCl pH 7.2, 10 mM EDTA, 2 mM EGTA, 250 mM sucrose, 1 mM DTT, 1 mM Benzamidine, 1 mM PMSF). The resulting material was transferred to a microtube, and centrifuged at 20,000 × *g* for 30 minutes at 4 °C. The supernatant was collected and the concentration of the extracted proteins was determined by the Bradford method [30]. As many inflammatory cytokines indicative of RA are expressed in the cartilage, ligament, pannus, articular capsule, and so forth, of CIA mice [31], we used these proteins to dissociate the selected phage clones from target antibodies by competitive elution.

### Purification and isolation of immunoglobulin G from sera

Immunoglobulin G (IgG) purification and isolation from sera of mice with acute arthritis and naïve mice was performed with Dynabeads® Protein G ( Invitrogen, Carlsbad, CA, USA), following the manufacturer’s instructions. Briefly, a total of 50 μl magnetic beads were washed twice with TBS-T 0.1 % (Tris-buffered saline: 50 mM Tris–HCl, 150 mM NaCl, pH 7.5 plus 0.1 % Tween 20) and then incubated with 100 μl pooled serum for 1 hour at room temperature. After binding, the bead–IgG complex was blocked with TBS plus 5 % bovine serum albumin (BSA) at 37 °C for 1 hour, washed three times with TBS-T 0.1 % and then resuspended in 200 μl TBS.

### Mimotope selection through phage display

For mimotope (phages expressing peptides on their surface) selection a PhD-12mer phage display peptide library kit (New England Biolabs, Beverly, MA, USA) was screened against IgG purified from CIA mice. This is a combinatorial library of random dodecapeptides fused to the N-terminus of the minor coat protein (pIII) of M13 phages. The library consists of 2.7 × 10^9^ diverse sequences that were amplified once to yield about 50 copies of each peptide sequence.

Based on scientific evidence showing that CIA mice and RA patients share several pathological features, circulating autoantibodies to common targets [29], and also because arthritis can be much easier to monitor in experimental animal models than in humans due to the complexity of the symptoms, we decided to perform the mimotope selection against IgG purified from CIA mice.

The strategy adopted for the mimotope selection consisted of a subtractive step to remove nonspecific phages by pre-incubating the phage peptide library with IgG purified from naïve mice. In order to remove nonspecific phages, a volume of 7 × 10^8^ beads/ml coupled with IgG purified from naïve mice serum was incubated with 1 × 10^11^ phage particles from the PhD-12 library in 200 μl TBS-T 0.1 % solution for 30 minutes at room temperature. After paramagnetic bead precipitation using the Magnetic Particle Concentrator (Dynal MPC™; Invitrogen), unbound phages were collected and incubated with beads coupled with IgG purified from CIA mice presenting signs of acute arthritis, following incubation for 30 minutes at room temperature. The unbound phages were discarded this time by washing ten times with TBS-T 0.1 %. For competitive elution, the bound phages were incubated for 30 minutes with 10 μg of the total protein extracted from inflamed joints of CIA mice. The eluted phages were amplified in *Escherichia coli* ER2738 strain (New England Biolabs) and purified by PEG-NaCl precipitation. After each of the three rounds of selection, individual bacterial colonies containing amplified phage clones were grown in a microtiter plate and titrated as described elsewhere [32].

### DNA extraction and sequencing

Phage DNA was isolated from 1 ml overnight cultures by precipitation with 1/6 volume PEG/NaI (20 % w/w, polyethylene glycol 8000) and iodide buffer (10 mm Tris–HCl (pH 8.0), 1 mm EDTA, and 4 m NaI). Phage DNA was precipitated with absolute ethanol, followed by a wash with 70 % ethanol, and resuspended in 20 μl Milli-Q water. Electrophoresis was performed on 0.8 % agarose gel stained with ethidium bromide solution in order to verify DNA quality. Sequencing reactions were carried out using the DyEnamic ET Dye Terminator Cycle Sequencing Kit (GE Healthcare, Pittsburg, PA, USA), with the primer −96 M13 (5’-OH CCC TCA TAG TTA GCG TAA CG-3’) following the manufacturer’s instructions, and detection was performed in a MegaBace 1000 Genetic Analyzer (Amersham Biosciences, Little Chalfont, UK) automatic capillary sequencer.

### Bioinformatic analysis

A tool that can be found on the Sequence Manipulation Suite collection of JavaScript programs [33] was utilized to obtain the reverse complementary sequences of the DNA extracted from phages. Amino acid sequences were deducted by ExPASy Proteomics and Sequence Analysis tool [34, 35]. For sequence similarity analysis, multiple sequence alignment was performed by ClustalW2 online server [36]. Three-dimensional structure prediction was performed using The Pepitope Server [37, 38], and Immune Epitope Database and Analysis Resource [39]. The PyMOL (available at [40]) plugin was used for showing the peptide surface. Antigenicity prediction was carried out using Kolaskar and Tongaonkar antigenicity scale [41, 42].

### Immunoreactivity of the selected mimotopes by phage-ELISA

For immunoreactivity measurements of selected mimotopes against sera from RA and HC groups, a phage-ELISA assay was performed. A 96-well Maxisorp™ microtiter plate (NUNC, New York, NY, USA) was coated in triplicate with anti-M13 monoclonal antibody (Amersham Biosciences) diluted (1:100) in carbonate buffer (0.1 M NaHCO_3_, pH 8.6) overnight at 4 °C. The plate was washed once with TBS-T 0.5 % and blocked for 1 hour at 37 °C with 5 % BSA diluted in TBS. Additionally, the plate was washed twice and incubated with culture supernatant containing amplified phage particles (~ 10^11^ pfu/μl) for 1 hour at 37 °C. The plate was washed three times followed by incubation with serum pools from the RA and HC groups diluted (1:100) in TBS-T 0.5 % plus 5 % BSA for 1 hour at 37 °C. The plate was washed three times more with TBS-T 0.5 % followed by incubation with HRP-conjugated rabbit anti-human IgG (Roche Applied Science, Indianapolis, IN, USA.) diluted (1:5,000) in TBS-T 0.5 % plus 5 % BSA for 1 hour at 37 °C. The ELISA plate was washed three times, revealed with OPD SigmaFast™ (Sigma-Aldrich) and read at 492 nm. The reactivity obtained by the wild-type M13 phage without displaying any peptide (performed for each sample tested) was used for data adjustment, where the final optical density (OD) values obtained for each mimotope were adjusted by subtracting the corresponding OD values obtained by the wild-type M13 phage.

### Purification of human anti-M12 antibody and M12 mimotope by immunoprecipitation

For purification of IgG antibodies that bind to the M12 mimotope, 1 × 10^11^ of M12 phage particles were covalently bound to Dynabeads® (Invitrogen). Thereafter, a volume of 50 μl of the solution containing the bead–M12 complex was separately incubated with 100 μl pooled sera from RA patients and HCs for 1 hour at room temperature under shaking for IgG:M12 binding. After incubation, the complex was washed ten times with TBS-T 0.1 % and the unbound nonspecific IgG present in the supernatant was discarded. The IgG that bound with high affinity to the M12 phage clone were eluted with 100 μl elution buffer (0.2 M Glycine-HCl, pH 2.2 and BSA 1 mg/ml) after incubation for 10 minutes at room temperature, followed by neutralization with 15 μl 1 M Tris–HCl (pH 9.1). The eluted IgG was incubated with 50 μl magnetic beads, as previous described. Subsequently, the solution containing the bead–IgG complex was incubated with 1 μg total protein extracted from inflamed joints of CIA mice for 1 hour at room temperature, under shaking condition for protein binding. The unbound proteins were discarded by washing ten times with TBS-T 0.1 %, followed by elution of the bound proteins with 100 μl 0.8 M acetic acid (pH 2.0). Proteins eluted were dried and submitted for MS analysis.

### Western blot analysis

Total proteins extracted from inflamed joints of CIA mice (1 μg) were separated by 10 % SDS-PAGE and transferred to a nitrocellulose membrane (GE Healthcare). The membrane was blocked for 1 hour with 3 % BSA in PBS, and then rinsed three times in washing buffer containing PBS-T 0.1 %. Thereafter, the membrane was incubated overnight at 4 °C with IgG purified from sera of RA patients and HCs diluted at 100 μg/ml in PBS plus 3 % BSA, washed three times and incubated for 1 hour with rabbit anti-human IgG conjugated with peroxidase (Roche Applied Science) diluted 1:5,000 in PBS plus 3 % BSA. The subsequent washing steps and detection procedures were performed according to the ECL Plus manual (GE Healthcare).

### Mass spectrometry

Protein digestion: dried proteins were suspended in 30 μl 0.2 % RapiGest and vortexed for 5 minutes. Protein digestion was carried out as described elsewhere with a few modifications [43]. Briefly, 10 μl 50 mM ammonium bicarbonate was added to the protein suspension to a final volume of 40 μl. Protein samples were denatured with 25 μl 0.2 % (w:v) RapiGest SF for 15 minutes at 80 °C, reduced with 2.5 μl 100 mM dithiothreitol at 60 °C for 30 minutes, alkylated with 2.5 μl 300 mM iodoacetamide at room temperature, and enzymatically digested at 37 °C overnight with trypsin at a 1:100 (w/w) enzyme to protein ratio. Then, 10 μl 5 % trifluoroacetic acid (TFA) was added to the digestion mixture to hydrolyze the RapiGest, and samples were incubated at 37 °C for 90 minutes. The tryptic peptide solution was then centrifuged at 14,000 rpm for 30 minutes at 6 °C, and the pH of the supernatant was adjusted to 2.6 by the addition of 10 μl 3 % acetonitrile 0.1 % formic acid.

Liquid chromatography/MS analysis: separation of peptides was performed using nanoliquid chromatography employing reverse phase. Peptides were injected into nanoLC through a nanoACQUITY system (Waters, Manchester, UK). Samples were first trapped in a Symmetry C18 5 μm, 180 mm × 20 mm column (Waters) with 0.1 % TFA in 3 % acetonitrile, then peptides were eluted from the trap column to an HSS T3 1.8 μm, 75 μm × 15 cm analytical column (Waters; mobile phase A, water with 0.1 % formic acid and B, 0.1 % formic acid in acetonitrile). Mass spectrometric acquisition was achieved in a Synapt MS Q-TOF mass spectrometer equipped with a NanoLockSpray source in the positive ion mode (Waters). For all measurements, the mass spectrometer was operated in the ‘V’ mode with a typical resolving power of at least 12,500. Data-independent scanning (MSE) experiments were performed by switching between low (3 eV) and elevated collision energies (15–50 eV) applied to the trap ‘T-wave’ cell filled with argon. Scan times of 0.8 s were used for low- and high-energy scans from m/z 50 to 2,000.

Protein identification and database analysis: protein identification was performed in Global Server v.2.5 (PLGS, Waters) with mouse UniProtKB Complete Proteome database. Up to one maximum missed cleavage by trypsin was allowed; fixed modification by carbamidomethylation (cysteine) and variable modifications by acetyl N-terminal and oxidation (methionine) were considered. The precursor and fragment ion mass error tolerances were adjusted to 10 and 20 ppm, respectively (default values). The criteria used for a positive protein match were at least three fragment ions per peptide, seven fragment ions per protein, and at least one peptide per protein hit. A false-positive discovery rate was allowed up to 4 %.

### Peptide design and synthesis

After bioinformatic analysis of the selected clones, the M12 peptide sequence was designed and chemically synthesized by GenScript USA Inc. (Piscataway, NJ, USA). The peptide was constructed with 17 residues (CNVNSKSPVERITGGGS), with amidation of the C-terminal and conjugation of the BSA to cysteine at the N-terminus, a design strategy to increase sensitivity and decrease crossreactivity on ELISA assay [44].

### Anti-M12 antibody detection by ELISA

A specific ELISA test was carried out to determine the M12 synthetic peptide reactivity to sera from patients with RA, SLE, AS and HCs. Ninety-six-well Maxisorp™ microtiter plates (NUNC) were coated with synthetic M12 (1.5 μg/ml) in carbonate buffer (0.1 M NaHCO_3_, pH 8.6), and incubated overnight at 4 °C. After blocking with 3 % BSA in PBS at 37 °C for 1 hour, 100 μl/well of sera from the different groups were diluted in blocking buffer (1:100) and incubated for 1 hour at 37 °C under gentle agitation. After four washes with PBS-T 0.05 %, HRP-conjugated rabbit anti-human IgG (Roche Applied Science) diluted (1:5,000) in the blocking buffer was incubated for 1 hour at 37 °C under gentle agitation. The ELISA plate was washed three times with PBS-T 0.05 %, revealed with OPD SigmaFast™ (Sigma-Aldrich) and read at 492 nm. All samples were tested in triplicate. The optimum point of reaction for anti-M12 antibody detection was determined using the receiver operating characteristic (ROC) curve, where a cut-off point was determined as the value of the parameter corresponding to the highest possible sensitivity without losing specificity. To calculate the ROC curve, sensitivity and specificity, we considered the control groups as a single group. Each serum sample was tested without M12 as negative control. The final OD values obtained for each RA, HC, SLE or AS samples were adjusted by subtracting the corresponding OD value obtained by the negative control. After data adjustment, OD values obtained for each sample from all groups were divided by the cut-off value for data normalization. The values obtained are expressed as reactivity index (RI), where samples presenting RI ≥1 were considered positives.

### Statistical analysis

Unpaired *t* test with Welch’s correction was used to evaluate the differences in sera reactivity in ELISA assays among groups for phage clones and the synthetic peptide. Sensitivity and specificity parameters were calculated based on the ROC curve analysis, and Fisher’s exact test was used for categorical data. To estimate the positive predictive accuracy, the area under the curve (AUC) was also determined. Pearson’s correlation was used for analysis among variables. All statistical analyses were performed using GraphPad Prism 5.0 software (GraphPad Software, Inc., San Diego, CA). *p* values less than 0.05 were considered statistically significant.

## Results

### Arthritis induction

**Fig. 1:**
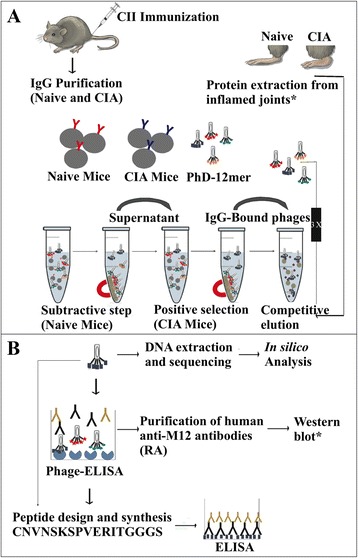
Schematic workflow illustrating the steps involved in the study. **a** Selection step, and **b** validation step. Arthritis was induced in collagen-induced arthritis (CIA) mice by immunization with type II collagen (CII). Immunoglobulin G (IgG) was purified by protein G beads from serum of mice presenting with acute arthritis and naïve mice. A phage display library was used to select mimotopes against purified IgG from CIA mice (three cycles of biopanning), with a subtractive step against IgG from naïve mice. IgG-bound phages from CIA mice were competitively eluted by incubation with total proteins extracted from inflamed joints. Total proteins extracted from inflamed joints were also used for anti-M12 antibody recognition in Western blot. Immunoreactivity of each selected mimotope was tested by phage exyme-linked immunosorbent assay (ELISA). After DNA extraction and in silico and in vitro analysis, the most reactive mimotope (M12) was identified as a peptide that mimics a predicted antigenic site of the human carbonic anhydrase III protein. Validation of the M12 synthetic peptide as a possible rheumatoid arthritis (RA) autoantigen was carried out by ELISA assay

Arthritis induction was efficient in CIA mice. A total of 80 % of male DBA/1 J mice developed acute arthritis around 60 days after CII immunization. The severity and incidence of arthritis were assessed as described in Methods. No manifestation of arthritis was observed in mice in the control group treated with PBS in CFA. A schematic workflow illustrating the steps employed in this study is shown in Fig. 1.

### Selection of mimotopes by phage display

**Fig. 2:**
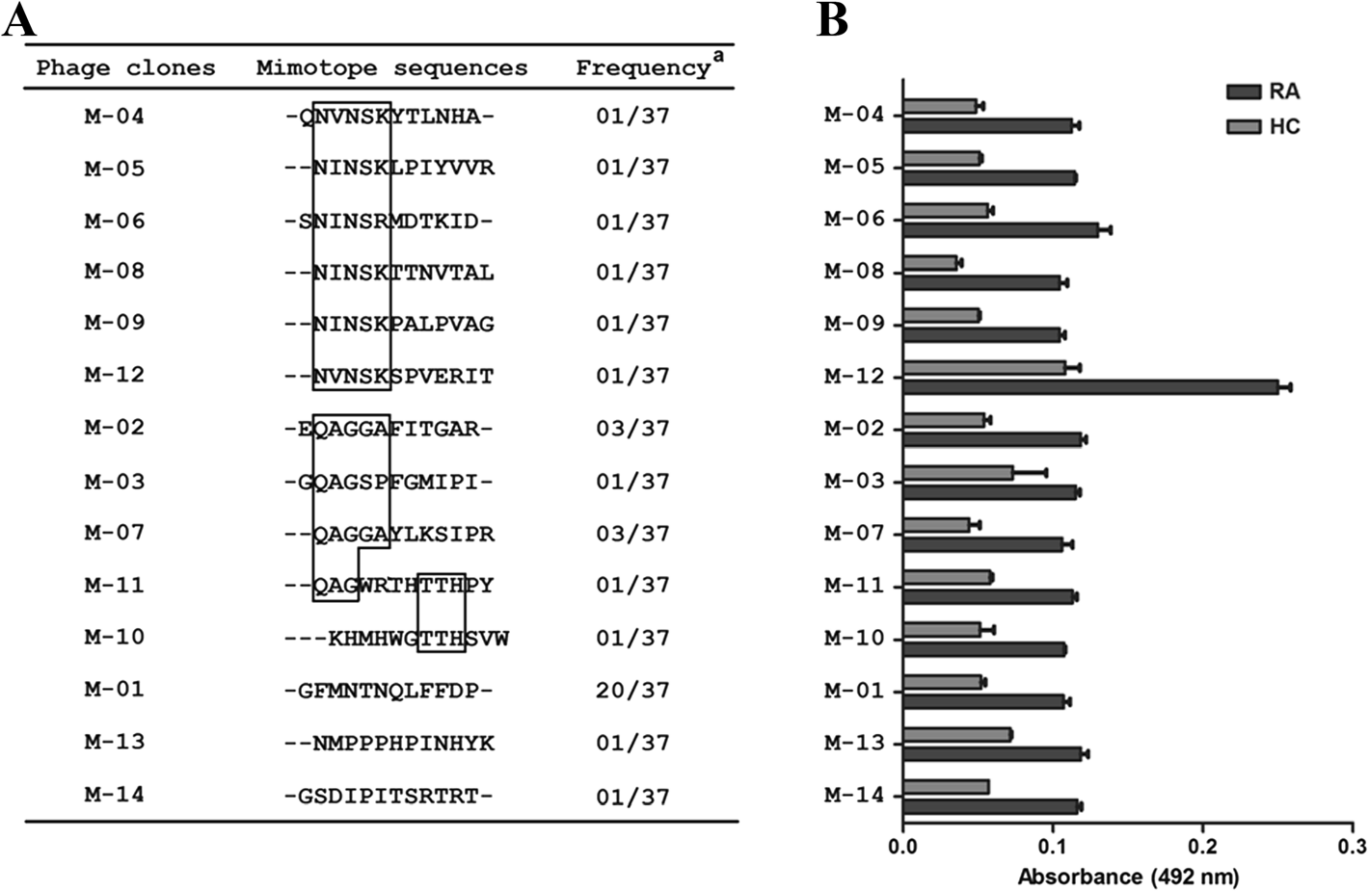
Perfomance of the mimotopes selected by phage display. **a** Multiple sequence alignment with all selected mimotopes showing their consensus sequences and frequency. **b** Reactivity obtained though the interaction of the mimotopes selected and pooled sera from rheumatoid arthritis (RA) patients and health controls (HC). ^a^Frequency is defined as the ratio of the number of phage clones expressing a common peptide sequence to that of the total phage clones obtained in the biopanning

The enrichment of phages recovered after each round of biopanning was determined by the output to input ratio. The increase from 8 × 10^4^ in the first round to 4 × 10^5^ after three rounds of affinity selection showed a clear enrichment of phage particles (Table 2). These data indicate a successful affinity selection of phages that specifically recognized IgG present in sera of DBA1/J mice with acute arthritis. A total of 37 randomly selected mimotopes were obtained after three rounds of biopanning using a phage displayed 12-mer random peptide library. From the 37 mimotopes selected, 14 presented different sequences. Alignment analysis revealed some consensus sequence between the mimotopes selected (Fig. 2a), indicating that these motifs were positively selected during the biopanning. The selected mimotopes showed different reactivities when tested against human sera by phage-ELISA, and all of them were able to discriminate RA patients from HCs. The reactivity values were very similar for all tested mimotopes, except for M12, which showed the highest values of absorbance (Fig. 2b) and the highest difference compared to the other clones (*p* < 0.05). Due to the higher reactivity compared to the other mimotopes, the common motif shared with another five clones, and the highest ratio of RA:HC, we focused on M12 peptide for further investigation. However, the other mimotopes selected will be explored in future studies.

**Table 2:** Enrichment of phage for each round of selection from phage display peptide library

	Number of phage particles		
Round	Input (cpu)	Output (cpu)	Ratio (output/input)
1st	1 × 10^11^	8 × 10^4^	8 × 10^−7^
2nd	1 × 10^11^	9 × 10^3^	9 × 10^−8^
3rd	1 × 10^11^	4 × 10^5^	4 × 10^−6^

*cpu* phage units

### Western blot analysis

**Fig. 3:**
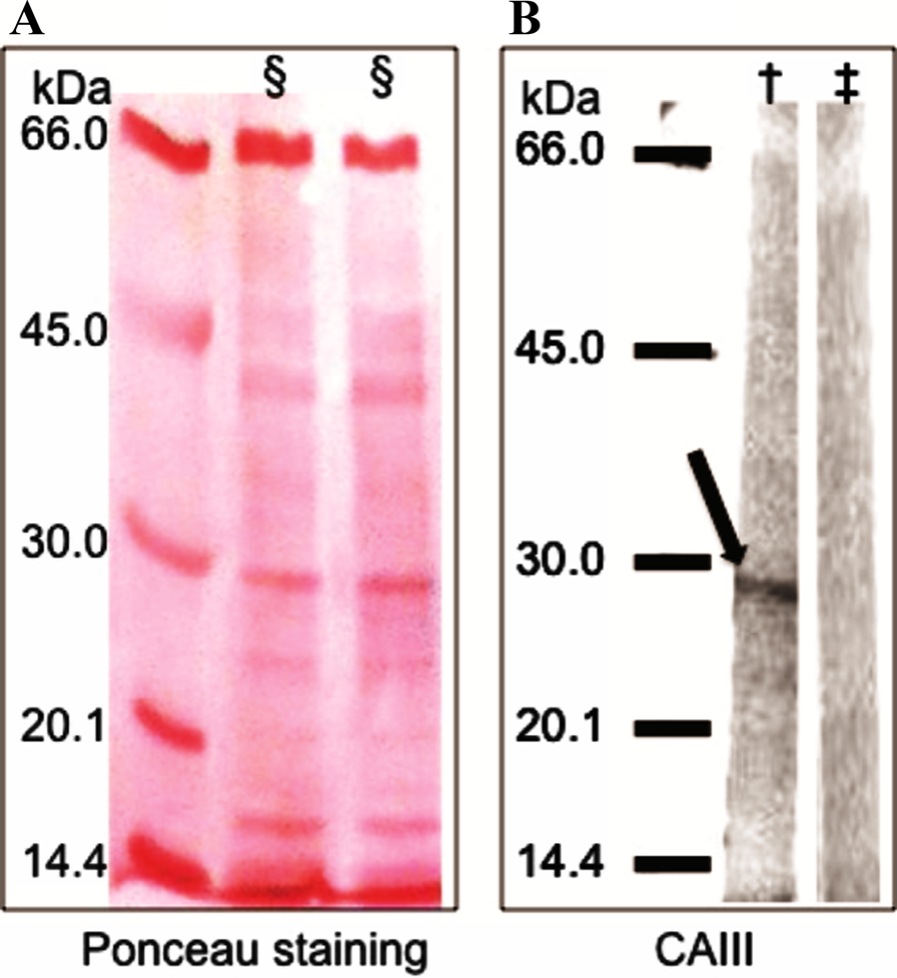
Western blot analyses. **a** The Ponceau staining shows 1 μg of total proteins extracted from inflamed joints of CIA mice (§) separated by 10 % SDS-PAGE. **b** The same membrane stained with Ponceau was probed with anti-M12 antibodies purified from pooled sera of RA patients (†) and HCs (‡). Anti-M12 antibodies displayed strong reactivity with a protein extracted from inflamed joints of CIA mice presenting with mass of approximately 29 kDa (*arrow*). Carbonic anhydrase III (CAIII) was identified as the protein target that is mimicked by the M12 mimotope. The membrane was cropped to allow differential incubation with anti-M12 antibodies purified from RA patients and HCs

SDS-PAGE fractionated proteins from inflamed joints of CIA mice were transferred to a nitrocellulose membrane. Western blot revealed that purified IgG from RA patients against the M12 mimotope recognized a protein with a molecular weight of approximately 29 kDa (Fig. 3).

### Antigen target and epitope prediction

MS analysis identified 15 putative proteins associated with anti-M12 IgG, where 14 proteins presented few peptide matches, with very low scores and sequence coverage and none of them presented a molecular mass close to 29 kDa. Therefore, based on the molecular weight of the protein target by anti-M12 antibodies observed in the Western blot results (29 kDa), the alignment of putative proteins with the M12 peptide sequence, and the sequencing coverage by MS, we have identified the carbonic anhydrase III (CAIII) [Uniprot/Swiss-Prot:P16015] as the protein target that the M12 peptide mimics. The CAIII protein showed 49.62 % of sequencing coverage, including the peptide sequence, which was aligned by conserved and semi-conserved amino acid (aa) residues between position 24–35 (KGDNQSPIELHT).

**Fig. 4:**
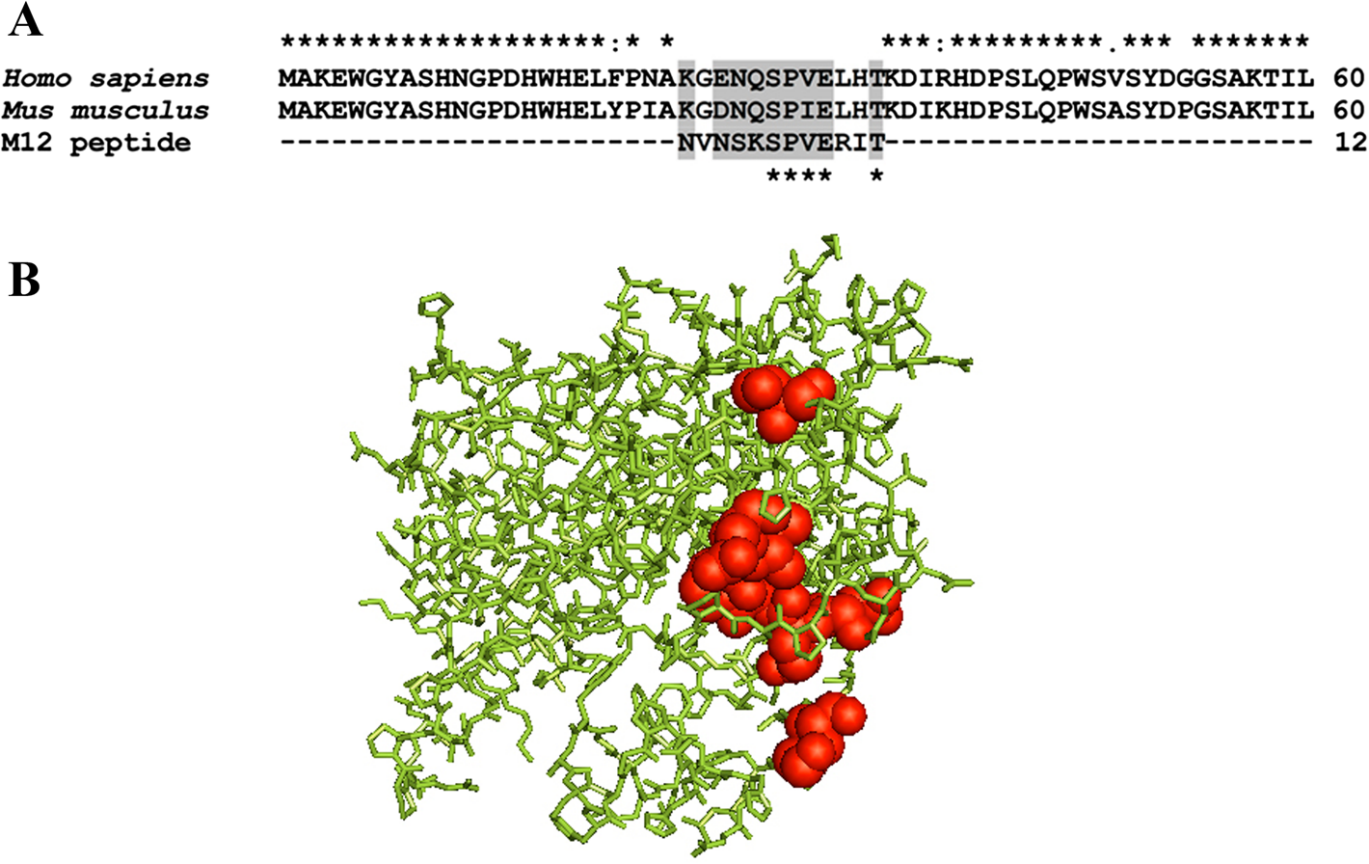
Peptide localization. **a** Multiple alignments of M12 peptide and deduced amino acid sequences of carbonic anhydrase III from *Homo sapiens* and *Mus musculus*. Conserved (*stars*) and semi-conserved residues (*gray*) are highlighted. Valine (V) amino acid is star marked because it matches a position between M12 peptide and human CAIII protein. **b** Model of the three-dimensional structure predicted in the PyMol server software for the human carbonic anhydrase III with M12 peptide localization

**Fig. 5:**
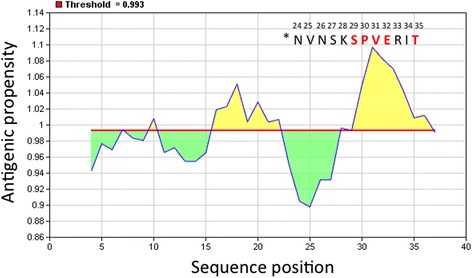
Antigenicity predictions using the Kolaskar-Tongaonkar algorithm. Threshold (0.993); average (0.993); minimum antigenicity (0.898); maximum antigenicity (1.097). Regions with antigenic propensity scale above 0.993 are antigenic. Window size and center position were 7 and 4, respectively. *Localization of M12 peptide in the CAIII protein (aa 24–35). Matched aa residues are presented in red.

The multiple sequence alignment revealed several homologous sequences between M12 peptide and the CAIII protein sequences from mouse and human. Nine (75 %) conserved or semi-conserved aa residues of the M12 peptide sequence matched a domain of the CAIII protein (Fig. 4a). A three-dimensional structural alignment was performed to predict the putative epitope site of the M12 peptide in the CAIII protein structure [PDB:3UYN], which confirmed its surface exposure and corroborated to the possible antibody-binding region in the external sequences of the predicted protein (Fig. 4b). Five (41.6 %) aa residues (SPVET) of the M12 peptide was matched to an antigenic region of the human CAIII protein (Fig. 5).

### Evaluation of the M12 peptide as a possible biomarker for RA diagnosis

**Fig. 6:**
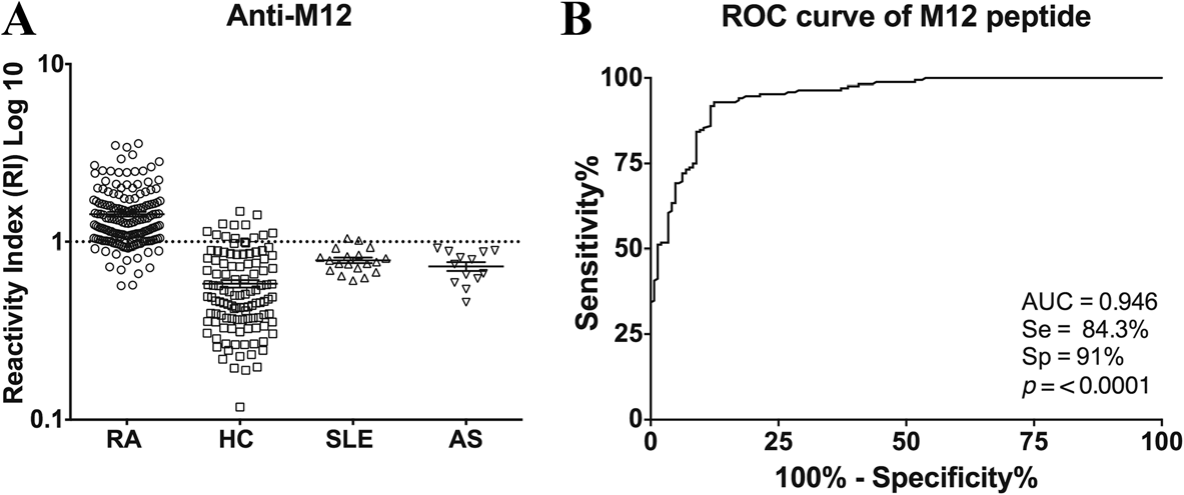
Anti-M12 antibody detection by ELISA. Detection of anti-M12 antibodies in sera from rheumatoid arthritis (RA) patients (n = 172), healthy controls (HC) (n = 113), systemic lupus erythematosus (SLE) patients (n = 19) and ankylosing spondylitis (AS) patients (n = 13) and the respectively receiver operating characteristic (ROC) curve. **a** Sera from the four groups individually tested for their ability to bind to synthetic M12 peptide. Horizontal line = cut-off value; error bars show mean and standard deviation. **b** ROC curve constructed based on the control groups. The area under the curve (AUC), sensitivity (Se), specificity (Sp) and corresponding *p*-value are indicated inside the graph

The M12 synthetic peptide was tested by ELISA with individual sera obtained from 172 RA patients, 113 HCs, 19 SLE patients and 13 AS patients to evaluate its potential as a diagnostic. The cut-off point was determined as the value of the parameter corresponding to the highest sensitivity without lowering the specificity. Of 172 RA patients, 145 (84.3 %) were positive to anti-M12 antibodies. The synthetic molecule was able to efficiently discriminate sera from RA patients and HCs (*p* < 0.0001), SLE (*p* < 0.0001) and AS (*p* < 0.0001) (Fig. 6a). The ROC curve analysis constructed based on the control groups was significant (*p* < 0.0001; AUC = 0.946). Based on the determined cut-off point, M12 synthetic peptide presented a specificity of 91 % and sensitivity of 84.3 % (Fig. 6b). For humoral immune response evaluation, 55 (32 %) serum samples from RA patients were simultaneously positive for RF, antiCCP and anti-M12 antibodies, while 45 (26.2 %) samples were positive only for anti-M12 and 12 (7 %) were negative for the three antibodies (Table 3).

**Table 3:** Antibodies positivity in the rheumatoid arthritis population

Antibody detection			Positivity	
Rheumatoid factor	AntiCCP	Anti-M12	n	%
+	−	−	8	4.6
−	+	−	0	0
−	−	+	45	26.2
+	+	−	7	4
+	−	+	32	18.6
−	+	+	13	7.6
+	+	+	55	32
−	−	−	12	7
Total			172	100

+ positive, − negative, *CCP* cyclic citrullinated peptide

### Correlation of anti-M12 humoral response with clinical variables

The weakly associated variables with anti-M12 levels from Gaussian populations (Pearson) analysis were disease duration (*r* = 0.1753) and the use of biological therapy (*r* = −0.2934) (Table 4).

**Table 4:** Clinical variables associated with detection of anti-M12 antibodies in patients with theumatoid arthritis

Variables	Anti-M12		
	Pearson *r*	*p*	*R* squared
Ethnicity	†	†	†
Gender	†	†	†
Age	†	†	†
Disease duration	0.1753	<0.05	0.03072
Rheumatoid factor	†	†	†
ESR	†	†	†
CRP	†	†	†
AntiCCP	†	†	†
Tender joints	†	†	†
Swollen joints	†	†	†
Biologic therapy	−0.2934	<0.005	0.08616
NSAIDs	†	†	†

Correlations were performed from Gaussian populations (Pearson) with a confidence interval of 95 %. ^†^Values not statistically significant. *CCP* cyclic citrullinated peptide, *CRP* C-reactive protein, *ESR* erythrocyte sedimentation rate, *NSAID* nonsteroidal anti-inflammatory drug

## Discussion

In order to identify new biomarkers for the improvement of RA diagnosis, we have used the CIA mouse model and PD technologies to select peptides that could mimic RA autoantigens. This approach allowed us to discover a new peptide with 12 amino acids (M12) that encompasses an antigenic domain of the CAIII protein. The use of the synthetic M12 peptide as an autoantigen in ELISA confirmed the presence of specific anti-M12 antibodies in sera of patients with RA with high specificity and sensitivity.

We have used the DBA1/J mouse as a model for inflammatory RA, where arthritis can be induced in susceptible mouse strains by immunization with CII [45]. DBA/1 J mice are CIA models of particular interest, since this strain is not known to have any immunologic aberrations or other pathologic defects [46]. CIA mice are widely used as an animal model for RA studies [14–17, 47–49]. We have chosen this experimental model due to the fact that genomic similarities between mice and humans are quite high, reaching 98 % [50]; it is much easier to monitor arthritis in mice than in humans, and also it is a unique experimental opportunity to increase our understanding of human arthritis. We confirmed the efficiency of the arthritis induction in our CIA mice strain, when a total of 80 % of DBA/1 J developed signs of arthritis around 60 days after CII immunization. We have used a subtractive PD selection against IgG present in sera of CIA mice that aims to select new RA biomarkers. We have chosen this technology due to its successful applications in biomarker discovery, especially when short random peptides are selected against specific target molecules, such as those selected against IgG present in sera for antigen discovery [51, 52].

The highly reactive peptide M12 was able to efficiently discriminate RA patients from HCs as well as from patients affected by SLE and AS, two other immune-mediated rheumatic diseases. In fact, the M12 peptide was not the most enriched mimotope after the rounds of phage amplification; however, the most frequently selected mimotope will not necessarily be the most reactive – selection is independent, which means that increasing one does not eliminate the effect of the other [53]. Many events during viral infection may contribute to increasing the amplification of specific phages. The biological reasons for abundances and growth advantages of some mimotopes have been discussed in many reports and include events like the phages ability to bind to bacterial pili, and interference with packing or infection [54–56]. The M12 peptide sequence was further characterized by reverse binding using its specific IgG antibodies to capture and identify its antigen target by MS, which led us to the CAIII protein. The CAIII protein is a member of a multigene family that encodes carbonic anhydrase isozymes. It is an abundant muscle protein and plays an important role in facilitated CO_2_ diffusion and diverse processes involving H^+^ and -HCO_3_ transport [57]. The CAIII gene encodes a protein of 260 aa and a molecular weight of approximately 29 kDa, which is well conserved throughout humans and mice. This protein has already been identified as an autoantigen expressed in the synovial membrane of RA patients [58] and its circulating autoantibodies have been found in many diseases [59]. The Kolaskar and Tongaonkar antigenicity scale is a semi-empirical method that makes use of physicochemical properties of aa residues and their frequencies of occurrence in experimentally known segmental epitopes, and is used to predict antigenic determinants on proteins. Using this parameter, this method that can predict antigenic determinants with 75 % accuracy [41] has been used in many studies [60–62]. Amino acid residues of the M12 peptide matched conservative and semi-conservative residues in a predicted antigenic and exposed site of a putative epitope of the human CAIII protein, which probably gave this peptide the ability to be recognized by specific IgG during the mimotope selection. This hypothesis is supported by the fact that semi-conserved aa residues present similar physicochemical properties with the original residue allowing antibody binding [63]. Of interest, a synthetic peptide between 24–54 aa residues of the human CAIII, that encompasses the M12 epitope region, has been commercially used to generate antiCAIII antibodies in rabbits (Product ID: ABIN391954). This fact probably reinforces the importance of the CAIII protein in RA and the potential use of this peptide as an antigen for antibody detection to aid in diagnosis.

A wide range of serum biomarkers has been assessed to improve diagnosis and prognosis of RA. However, only the RF and antiCCP antibodies have gained wide acceptance [10]. The use of recombinant CAIII protein as an antigen has confirmed the presence of specific antiCAIII antibodies in RA sera [58, 59]; however, its sensitivity considering the entire CAIII protein was only 17 %. In fact, when compared with HCs, the specificity of circulating autoantibodies was high (100 %), but reached only medium to low specificity when compared with other autoimmune diseases, such as SLE (67 %) [58]. The crossreactivity and lack of sensitivity of the whole CAIII protein suggests the presence of multiple epitopes sharing common regions with other proteins, as well as the presence of immunodominant epitopes that surpass the response to the M12 critical epitope, which may have generated either false positive results or an insufficient reactivity. On the other hand, the use of the M12 peptide conferred more sensitivity and specificity in the detection of RA, since it was able to discriminate RA patients from HCs, and patients with AS and SLE, with high accuracy.

Several studies have measured the three different generations of antiCCP antibodies in RA patients in comparison with control groups. These antibodies present specificities in the range of 65 % to 100 % (mean of 93.4 %) and sensitivities in the range of 42 % to 92.2 % (mean of 64.3 %) [9, 64–71]. RF testing presents specificities in the range 85 % to 90.29 % and sensitivities in the range 46.26 % to 90 % [72–74]. The diagnostic parameters for the M12 peptide for RA diagnosis presented specificity of 91 % and sensitivity of 84.3 %, which is very close to the mean of values reported for antiCCP and RF antibodies, the two most currently used serum biomarkers for RA. On the other hand, anti-M12 antibodies were detected in 26.2 % of samples that are negative for antiCCP and RF. We speculate that the high percentage of positive samples for anti-M12 antibodies might be due to the different tests used to cut-off calculation, or even the patients disease activity at the time of sample collection or individual response to therapy. In this context, high levels of antiCCP and RF antibodies in RA patients have already been associated with an insufficient response to therapy [75, 76], while anti-M12 antibodies were weakly associated with therapeutic response in our RA cohort, which may help to explain the percentage of positive samples only for M12. It is widely accepted that the identification of RA at early stages and consequently the implementation of effective treatment strategies can significantly improve patient prognosis [77]. Anti-M12 antibodies showed a weak association with disease duration, suggesting its potential use as a biomarker in different stages during the process of RA development.

## Conclusions

We have selected and identified a peptide that is capable of detecting specific circulating IgG in the serum of RA patients with high specificity and sensitivity. Although antibodies against synthetic M12 peptide were detected in patients with early and established RA, its potential use in the diagnosis at different stages of the disease remains to be studied during follow-up in a well characterized cohort of patients with early RA, and also its specificity as a serum biomarker should be further studied in a much larger cohort of patients with other inflammatory diseases. This peptide mimics a predicted antigenic region of the human carbonic anhydrase III by linear sequence analysis and could be used as an antigen for detection of specific RA autoantibodies.

## Abbreviations

Aa: Amino acid
ACPA: Anti-Citrullinated peptide antibody
AS: Ankylosing spondylitis
AUC: Area under the curve
BSA: Bovine serum albumin
CAIII: Carbonic anhydrase III
CCP: Cyclic citrullinated peptides
CFA: Complete Freund’s adjuvant
CIA: Collagen-induced arthritis
CII: Type II collagen
CRP: C-reactive protein
ELISA: Enzyme-linked immunosorbent assay
ESR: Erythrocyte sedimentation rate
HC: Healthy control
IgG: Immunoglobulin G
MS: Mass spectrometry
OD: Optical density
PBS: Phosphate-buffered saline
PD: Phage display
RA: Rheumatoid arthritis
RF: Rheumatoid factor
RI: Reactivity index
ROC: Receiver operating characteristic
SLE: Systemic lupus erythematosus
TBS: Tris-buffered saline
TFA: Trifluoroacetic acid

## References

[1] Rindfleisch JA, Muller D (2005). Diagnosis and management of rheumatoid arthritis. Am Fam Physician..

[2] National Collaborating Centre for Chronic Conditions (UK) (2009). Rheumatoid Arthritis: National Clinical Guideline for Management and Treatment in Adults.

[3] van Venrooij WJ, van Beers JJ, Pruijn GJ (2011). Anti-CCP antibodies: the past, the present and the future. Nat Rev Rheumatol..

[4] van Venrooij WJ, Hazes JM, Visser H (2002). Anticitrullinated protein/peptide antibody and its role in the diagnosis and prognosis of early rheumatoid arthritis. Neth J Med..

[5] Rantapaa-Dahlqvist S (2005). Diagnostic and prognostic significance of autoantibodies in early rheumatoid arthritis. Scand J Rheumatol..

[6] Schellekens GA, Visser H, de Jong BA, van den Hoogen FH, Hazes JM, Breedveld FC (2000). The diagnostic properties of rheumatoid arthritis antibodies recognizing a cyclic citrullinated peptide. Arthritis Rheum..

[7] Coenen D, Verschueren P, Westhovens R, Bossuyt X (2007). Technical and diagnostic performance of 6 assays for the measurement of citrullinated protein/peptide antibodies in the diagnosis of rheumatoid arthritis. Clin Chem..

[8] Vander Cruyssen B, Cantaert T, Nogueira L, Clavel C, De Rycke L, Dendoven A (2006). Diagnostic value of anti-human citrullinated fibrinogen ELISA and comparison with four other anti-citrullinated protein assays. Arthritis Res Ther..

[9] Goeb V, Jouen F, Gilbert D, Le Loet X, Tron F, Vittecoq O (2009). Diagnostic and prognostic usefulness of antibodies to citrullinated peptides. Joint Bone Spine..

[10] Taylor P, Gartemann J, Hsieh J, Creeden J (2011). A systematic review of serum biomarkers anti-cyclic citrullinated peptide and rheumatoid factor as tests for rheumatoid arthritis. Autoimmune Dis..

[11] van der Helm-van Mil AH, Verpoort KN, Breedveld FC, Toes RE, Huizinga TW (2005). Antibodies to citrullinated proteins and differences in clinical progression of rheumatoid arthritis. Arthritis Res Ther..

[12] Syversen SW, Gaarder PI, Goll GL, Odegard S, Haavardsholm EA, Mowinckel P (2008). High anti-cyclic citrullinated peptide levels and an algorithm of four variables predict radiographic progression in patients with rheumatoid arthritis: results from a 10-year longitudinal study. Ann Rheum Dis..

[13] Mjaavatten MD, van der Heijde DM, Uhlig T, Haugen AJ, Nygaard H, Bjorneboe O (2011). Should anti-citrullinated protein antibody and rheumatoid factor status be reassessed during the first year of followup in recent-onset arthritis? A longitudinal study. J Rheumatol..

[14] Park KH, Mun CH, Kang MI, Lee SW, Lee SK, Park YB (2015). Treatment of collagen-induced arthritis using immune modulatory properties of human mesenchymal stem cells. Cell Transpl..

[15] Chow LN, Choi KY, Piyadasa H, Bossert M, Uzonna J, Klonisch T (2014). Human cathelicidin LL-37-derived peptide IG-19 confers protection in a murine model of collagen-induced arthritis. Mol Immunol..

[16] Eneljung T, Tengvall S, Jirholt P, Henningsson L, Holmdahl R, Gustafsson K (2013). Antigen-specific gene therapy after immunisation reduces the severity of collagen-induced arthritis. Clin Dev Immunol..

[17] Ding X, Hu J, Li J, Zhang Y, Shui B, Ding Z (2014). Metabolomics analysis of collagen-induced arthritis in rats and interventional effects of oral tolerance. Anal Biochem..

[18] Rosloniec EF, Cremer M, Kang AH, Myers LK, Brand DD (2010). Collagen-induced arthritis. Curr Protoc Immunol..

[19] Wang M, Li X, Chen J, Zhou Y, Cao H, Wu X (2011). Screening and evaluating the mimic peptides as a useful serum biomarker of ankylosing spondylitis using a phage display technique. Rheumatol Int..

[20] Sergeeva A, Kolonin MG, Molldrem JJ, Pasqualini R, Arap W (2006). Display technologies: application for the discovery of drug and gene delivery agents. Adv Drug Deliv Rev..

[21] Samoylova TI, Morrison NE, Globa LP, Cox NR (2006). Peptide phage display: opportunities for development of personalized anti-cancer strategies. Anticancer Agents Med Chem..

[22] Alves PT, Fujimura PT, Morais LD, Goulart LR (2014). Revisiting the CD14: epitope mapping by phage display. Immunobiology..

[23] Reis CF, Carneiro AP, Vieira CU, Fujimura PT, Morari EC, Silva SJ (2013). An antibody-like peptide that recognizes malignancy among thyroid nodules. Cancer Lett..

[24] Santos PS, Sena AA, Nascimento R, Araujo TG, Mendes MM, Martins JR (2013). Epitope-based vaccines with the Anaplasma marginale MSP1a functional motif induce a balanced humoral and cellular immune response in mice. PLoS One..

[25] Scott JK, Smith GP (1990). Searching for peptide ligands with an epitope library. Science..

[26] Huang J, Ru B, Dai P (2011). Bioinformatics resources and tools for phage display. Molecules..

[27] Somers K, Geusens P, Elewaut D, De Keyser F, Rummens JL, Coenen M (2011). Novel autoantibody markers for early and seronegative rheumatoid arthritis. J Autoimmun..

[28] Aletaha D, Neogi T, Silman AJ, Funovits J, Felson DT, Bingham CO (2010). 2010 rheumatoid arthritis classification criteria: an American College of Rheumatology/European League Against Rheumatism collaborative initiative. Ann Rheum Dis..

[29] Brand DD, Latham KA, Rosloniec EF (2007). Collagen-induced arthritis. Nat Protoc..

[30] Bradford MM (1976). A rapid and sensitive method for the quantitation of microgram quantities of protein utilizing the principle of protein-dye binding. Anal Biochem..

[31] O’Valle F, Peregrina M, Crespo-Lora V, Galindo-Moreno P, Roman M, Padial-Molina M, et al. Osteoarticular expression of Musashi-1 in an Experimental model of arthritis. Biomed Res Int. 2015; Article ID 681456, in press. 10.1155/2015/681456 PMC4433648 26064941

[32] BD Barbas CF, Scott JK, Silverman GJ (2001). Phage display: a laboratory manual.

[33] Stothard P (2000). The sequence manipulation suite: JavaScript programs for analyzing and formatting protein and DNA sequences. Biotechniques..

[34] Artimo P, Jonnalagedda M, Arnold K, Baratin D, Csardi G, de Castro E (2012). ExPASy: SIB bioinformatics resource portal. Nucleic Acids Res..

[35] ExPASy Proteomics and Sequence Analysis tool. http://web.expasy.org/translate/.

[36] ClustalW2 online server. http://www.ebi.ac.uk/Tools/msa/clustalw2/.

[37] Mayrose I, Penn O, Erez E, Rubinstein ND, Shlomi T, Freund NT (2007). Pepitope: epitope mapping from affinity-selected peptides. Bioinformatics..

[38] The Pepitope Server. http://pepitope.tau.ac.il/.

[39] Immune Epitope Database and Analysis Resource. http://www.iedb.org/.

[40] Pymol. http://www.pymol.org.

[41] Kolaskar AS, Tongaonkar PC (1990). A semi-empirical method for prediction of antigenic determinants on protein antigens. FEBS Lett..

[42] Kolaskar and Tongaonkar antigenicity scale. http://tools.immuneepitope.org/bcell/.

[43] Camargo M, Intasqui Lopes P, Del Giudice PT, Carvalho VM, Cardozo KH, Andreoni C (2013). Unbiased label-free quantitative proteomic profiling and enriched proteomic pathways in seminal plasma of adult men before and after varicocelectomy. Hum Reprod..

[44] Casey JL, Coley AM, Street G, Parisi K, Devine PL, Foley M (2006). Peptide mimotopes selected from a random peptide library for diagnosis of Epstein-Barr virus infection. J Clin Microbiol..

[45] Williams RO (2007). Collagen-induced arthritis in mice: a major role for tumor necrosis factor-alpha. Methods Mol Biol..

[46] Corthay A, Hansson AS, Holmdahl R (2000). T lymphocytes are not required for the spontaneous development of entheseal ossification leading to marginal ankylosis in the DBA/1 mouse. Arthritis Rheum..

[47] Eros G, Ibrahim S, Siebert N, Boros M, Vollmar B (2009). Oral phosphatidylcholine pretreatment alleviates the signs of experimental rheumatoid arthritis. Arthritis Res Ther..

[48] Chiba A, Mizuno M, Tomi C, Tajima R, Alloza I, di Penta A (2012). A 4-trifluoromethyl analogue of celecoxib inhibits arthritis by suppressing innate immune cell activation. Arthritis Res Ther..

[49] Hong SH, Kwone JT, Lee JH, Lee S, Lee AY, Cho WY (2014). Ascidian tunicate extracts attenuate rheumatoid arthritis in a collagen-induced murine model. Nat Prod Commun..

[50] Mural RJ, Adams MD, Myers EW, Smith HO, Miklos GL, Wides R (2002). A comparison of whole-genome shotgun-derived mouse chromosome 16 and the human genome. Science..

[51] Weng X, Liao Q, Li K, Li Y, Mi M, Zhong D (2012). Screening serum biomarker of knee osteoarthritis using a phage display technique. Clin Biochem..

[52] Costa LE, Lima MI, Chavez-Fumagalli MA, Menezes-Souza D, Martins VT, Duarte MC (2014). Subtractive phage display selection from canine visceral leishmaniasis identifies novel epitopes that mimic Leishmania infantum antigens with potential serodiagnosis applications. Clin Vaccine Immunol..

[53] Derda R, Tang SK, Li SC, Ng S, Matochko W, Jafari MR (2011). Diversity of phage-displayed libraries of peptides during panning and amplification. Molecules..

[54] Kuzmicheva GA, Jayanna PK, Sorokulova IB, Petrenko VA (2009). Diversity and censoring of landscape phage libraries. Protein Eng Des Sel..

[55] Rodi DJ, Soares AS, Makowski L (2002). Quantitative assessment of peptide sequence diversity in M13 combinatorial peptide phage display libraries. J Mol Biol..

[56] Rodi DJ, Makowski L (1997). Structure and Function of Macromolecular Assembly.

[57] Edwards YH, Tweedie S, Lowe N, Lyons G (1992). Carbonic anhydrase 3 (CA3), a mesodermal marker. Symp Soc Exp Biol..

[58] Robert-Pachot M, Desbos A, Moreira A, Becchi M, Tebib J, Bonnin M (2007). Carbonic anhydrase III: a new target for autoantibodies in autoimmune diseases. Autoimmunity..

[59] Liu C, Wei Y, Wang J, Pi L, Huang J, Wang P (2012). Carbonic anhydrases III and IV autoantibodies in rheumatoid arthritis, systemic lupus erythematosus, diabetes, hypertensive renal disease, and heart failure. Clin Dev Immunol..

[60] Srinivasan P, Kumar SP, Karthikeyan M, Jeyakanthan J, Jasrai YT, Pandya HA (2011). Epitope-based immunoinformatics and molecular docking studies of nucleocapsid protein and ovarian tumor domain of crimean-congo hemorrhagic fever virus. Front Genet..

[61] Ranjbar MM, Ghorban K, Alavian SM, Keyvani H, Dadmanesh M, Roayaei Ardakany A (2013). GB virus C/hepatitis G virus envelope glycoprotein E2: computational molecular features and immunoinformatics study. Hepat Mon..

[62] Sealey KL, Kirk RS, Walker AJ, Rollinson D, Lawton SP (2013). Adaptive radiation within the vaccine target tetraspanin-23 across nine Schistosoma species from Africa. Int J Parasitol..

[63] Ng PC, Henikoff S (2006). Predicting the effects of amino acid substitutions on protein function. Annu Rev Genomics Hum Genet..

[64] Forslind K, Ahlmen M, Eberhardt K, Hafstrom I, Svensson B, Group BS (2004). Prediction of radiological outcome in early rheumatoid arthritis in clinical practice: role of antibodies to citrullinated peptides (anti-CCP). Ann Rheum Dis..

[65] Herold M, Boeser V, Russe E, Klotz W (2005). Anti-CCP: history and its usefulness. Clin Dev Immunol..

[66] Wagner E, Skoumal M, Bayer PM, Klaushofer K (2009). Antibody against mutated citrullinated vimentin: a new sensitive marker in the diagnosis of rheumatoid arthritis. Rheumatol Int..

[67] Mutlu N, Bicakcigil M, Tasan DA, Kaya A, Yavuz S, Ozden AI (2009). Comparative performance analysis of 4 different anti-citrullinated protein assays in the diagnosis of rheumatoid arthritis. J Rheumatol..

[68] Fernandez-Suarez A, Reneses S, Wichmann I, Criado R, Nunez A (2005). Efficacy of three ELISA measurements of anti-cyclic citrullinated peptide antibodies in the early diagnosis of rheumatoid arthritis. Clin Chem Lab Med..

[69] Kudo-Tanaka E, Ohshima S, Ishii M, Mima T, Matsushita M, Azuma N (2007). Autoantibodies to cyclic citrullinated peptide 2 (CCP2) are superior to other potential diagnostic biomarkers for predicting rheumatoid arthritis in early undifferentiated arthritis. Clin Rheumatol..

[70] van Gaalen FA, Visser H, Huizinga TW (2005). A comparison of the diagnostic accuracy and prognostic value of the first and second anti-cyclic citrullinated peptides (CCP1 and CCP2) autoantibody tests for rheumatoid arthritis. Ann Rheum Dis..

[71] Kim S, Kim JH, Lee JH, Kim HS (2010). Evaluation of three automated enzyme immunoassays for detection of anti-cyclic citrullinated peptide antibodies in qualitative and quantitative aspects. Rheumatology..

[72] Binesh F, Salehabadi HS, Behniafard N, Ranginkaman K, Behniafard N (2014). A comparative assessment of the diagnostic value of anti-cyclic citrullinated peptide antibodies and rheumatoid factor in rheumatoid arthritis. J Clin Exp Pathol..

[73] Nishimura K, Sugiyama D, Kogata Y, Tsuji G, Nakazawa T, Kawano S (2007). Meta-analysis: diagnostic accuracy of anti-cyclic citrullinated peptide antibody and rheumatoid factor for rheumatoid arthritis. Ann Intern Med..

[74] Nell VP, Machold KP, Stamm TA, Eberl G, Heinzl H, Uffmann M (2005). Autoantibody profiling as early diagnostic and prognostic tool for rheumatoid arthritis. Ann Rheum Dis..

[75] Visser K, Goekoop-Ruiterman YP, de Vries-Bouwstra JK, Ronday HK, Seys PE, Kerstens PJ (2010). A matrix risk model for the prediction of rapid radiographic progression in patients with rheumatoid arthritis receiving different dynamic treatment strategies: post hoc analyses from the BeSt study. Ann Rheum Dis..

[76] Mikuls TR, O’Dell JR, Stoner JA, Parrish LA, Arend WP, Norris JM (2004). Association of rheumatoid arthritis treatment response and disease duration with declines in serum levels of IgM rheumatoid factor and anti-cyclic citrullinated peptide antibody. Arthritis Rheum..

[77] Vermeer M, Kuper HH, Hoekstra M, Haagsma CJ, Posthumus MD, Brus HL (2011). Implementation of a treat-to-target strategy in very early rheumatoid arthritis: results of the Dutch Rheumatoid Arthritis Monitoring remission induction cohort study. Arthritis Rheum..

